# Can pathway-specific LFPs be obtained in cytoarchitectonically complex structures?

**DOI:** 10.3389/fnsys.2014.00066

**Published:** 2014-05-01

**Authors:** Julia Makarova, Tania Ortuño, Alejandra Korovaichuk, Javier Cudeiro, Valeri A. Makarov, Casto Rivadulla, Oscar Herreras

**Affiliations:** ^1^Department of Systems Neuroscience, Cajal Institute—CSICMadrid, Spain; ^2^Group of Neuroscience and Motor Control (NEUROcom) and Institute for Biomedical Research of A Coruña, University of A CoruñaLa Coruña, Spain; ^3^Department of Applied Mathematics, Universidad Complutense de MadridMadrid, Spain

**Keywords:** local field potentials, spatial discrimination, independent component analysis, spontaneous activity, network activity, lateral geniculate nucleus, multicompartmental neuron model, LFP model

## Abstract

Deciphering how the brain encodes the continuous flow of information contained in natural stimuli requires understanding the spontaneous activity of functional assemblies in multiple neuronal populations. A promising integrative approach that combines multisite recordings of local field potentials (LFP) with an independent component analysis (ICA) enables continuous readouts of population specific activities of functionally different neuron groups to be obtained. We previously used this technique successfully in the hippocampus, a single-layer neuronal structure. Here we provide numerical evidence that the cytoarchitectonic complexity of other brain structures does not compromise the value of the ICA-separated LFP components, given that spatial sampling of LFP is representative. The spatial distribution of an LFP component may be quite complex due to folded and multilayered structure of the neuronal aggregate. Nevertheless, the time course of each LFP component is still a reliable postsynaptic convolution of spikes fired by a homogeneous afferent population. This claim is supported by preliminary experimental data obtained in the lateral geniculate nucleus of the awake monkey.

## Local field potentials in simple and complex structures

The coding of information and its transfer across neural circuits is largely based on synchronized spike activity generated by functional neural assemblies rather than individual neurons. Part of this electrical activity is reflected in local field potentials (LFPs), a postsynaptic convolution of spikes from afferent neurons. Thus, LFPs mirror the fluctuations of ongoing activity in multiple local and remote upstream assemblies of neurons. Varying co-activation of assemblies in different structures projecting to the same recording site produces complex mixed LFP activity there. Accordingly, LFPs can have different values and even polarity at two nearby locations. Therefore, time fluctuations of raw LFPs do not provide particularly useful information, except for the few cases when only one input is at work (i.e., pathway-specific LFPs). Thus, recording LFPs with a single electrode hardly ever proves to be useful to gain information regarding their cellular correlates. Rather, it is necessary to record simultaneously over a large area that covers the extension of the postsynaptic neurons generating LFPs and attempt to discriminate the mixed inputs from the spatial distribution of their respective postsynaptic electric fields.

The contemporary literature on LFPs in the cortex reflects the strong focus on issues such as the spatial reach of currents from their sources, frequency-dependent propagation, the reliability and mechanisms of long-distance synchronization, and the contribution of local spikes or other cellular and non-cellular sources (Lindén et al., 2011; Buzsáki et al., 2012). LFPs are much larger in cytoarchitectonically simpler structures such as the hippocampus and these issues are addressed less often as they become less relevant or easily manageable. Thus, ordered structures may help to overcome the main technical problem initially identified by the pioneers of LFP, namely the spatial mixing of multiple sources (Lorente de Nó, 1947; Purpura, 1959; for a comprehensive discussion of this issue see Elul, 1972). Indeed, while the physical foundations of LFPs are well known (Nunez and Srinivasan, 2006), the complex geometry of the sources of these potentials in the brain cast important technical limitations to understand their cellular basis. To cut a long story short, the trans-membrane currents of single neurons contribute to LFPs and hence, their synchronization is necessary for the summation of analogous currents in the extracellular space. Thus, it is also clear that individual neurons should be arranged so that their individual currents can add. Indeed, spatial factors ultimately determine the amplitude, polarity and extension of such compound electrical fields. The most influential factors are the geometry of individual neurons, their three-dimensional arrangement, and the distribution of activating synaptic inputs on individual cells and the population as a whole. The variable contributions and combinations of these factors in different brain structures make LFPs large or small, positive or negative, and they may reflect active or passive synaptic currents, reaching locations far from the source or remaining local, and they may or may not be related to the afferent input (Benito et al., 2013; Fernández-Ruiz et al., 2013).

Given the complex geometry of neurons, which makes their surrounding fields too complex, the amount of microscopic information required to reliably calculate the amplitude and sign of an LFP is beyond possibilities of experimental studies. However, acceptable estimates may be obtained for naturally occurring population activity of neuron assemblies. If target neurons are arranged regularly, their synchronous activation facilitates the spatial segregation of inward and outward post-synaptic currents in the extracellular space. This leads to large field potentials with a simplified spatial distribution, and it is also the basis for customary evoked potentials in laminar structures such as the hippocampus, cortex and cerebellum. The spatial distribution of these fields facilitates their association to electrogenic membrane events with subcellular accuracy. However, activation is far less synchronous during spontaneous activity and it may have a patchy coverage of the target populations, typically engaging multiple afferent pathways. Although smaller and mixed in with others, the electric field produced by activation of each pathway maintains a spatial distribution. Indeed, this is a feature that spatial discrimination techniques can take advantage of to separate the pathway-specific contributions to LFPs (Makarov et al., 2010), such as the independent component analysis (ICA; Bell and Sejnowski, 1995). Here we show that this technique may also be useful in other cytoarchitectonically complex irregular structures.

The regularity of simple neural structures helped us to validate the ICA as a tool to identify and quantify the anatomical pathways contributing to hippocampal LFPs. The limited number of components the ICA may retrieve forces the myriad microscopic inputs activated over the time to group into a few components that preserve a common spatial distribution, which happens to be those corresponding to the natural anatomical pathways terminating on specific dendritic domains of target populations. Occasional co-activation of two or more pathways with partially overlapped synaptic territories is efficiently segregated by the ICA (Makarova et al., 2011). Before we can use the time fluctuations of an ICA-derived LFP component in a quantitative manner, its pathway-specificity must be proven. This can be explored through cross-checking with additional data, such as correlation with spikes in the population of origin, postsynaptic pharmacology, modulation of afferent populations, matching to evoked activity and to anatomical data (Korovaichuk et al., 2010; Fernández-Ruiz et al., 2012: for a detailed explanation on the limitations and practical hints for their solution, see Makarova et al., 2011; Benito et al., 2013). In the hippocampus we reported that all but one of the major ICA-derived components are pathway-specific and correspond to known local and distant afferent excitatory and inhibitory populations (Korovaichuk et al., 2010; Benito et al., 2013).

## The clue is the dissociation of spatial and temporal parts of LFPs

To understand why the ICA can be applied to separate pathway-specific LFP components in any structure it is necessary to envisage what it does with the specific signals and the spatiotemporal nature of these. The ICA operates on spatio-temporal matrices (for LFPs: the recording sites vs. time samples of voltage fluctuations), and it returns a collection of LFP components, presumably the original signals linearly “blended” in the volume. Importantly, these components have separated spatial and temporal elements. The spatial part is formed by a set of weight-electrode values that jointly outline a spatial distribution curve. Such curves can be compared to anatomical landmarks when the placement of the electrodes is known, and they help to localize the activated synaptic territories. The temporal part contains the fluctuations of the source over time (for components of LFPs: how many synapses/target neurons are activated over time), and is no longer dependent on the activity of electrical sources in other sites.

There are numerous ICA algorithms and is always advisable to test a few on the signals of interest (Calhoun et al., 2001; Stone et al., 2002). In our hands, the ICA algorithms that perform best for LFPs are those that maximize the spatial coherence of the sources. This is explained by the fact that bombardment by individual fibers spans the entire synaptic territory of a given projection over time and thus, the spatial distribution curves obtained over sufficiently long epochs are stable and they match the field produced by activation of the entire pathway well. By contrast, ICA algorithms that maximize independence based on temporal or hybrid spatio-temporal features of LFPs return severely cross-contaminated components that deviate from the original sources. Indeed, even if certain temporal patterns are characteristic of a given afferent population, their use to separate LFP components violates the instantaneous nature of electric fields and an uncontaminated separation is not guaranteed.

Although, ICA has been proven to be efficient in the regular structure of the hippocampus, there appears to be some skepticism in extending this technique to other less regular brain structures. However, such a view has no biophysical grounding and rather, it reflects confusion as to how ICA deals with signals composed of static sources with similar temporal patterns. In practice this is not really a problem since small time lags between sources, or the use of sufficiently long epochs for analysis, minimizes temporal cross-contamination by maximizing spatial reliability (Makarova et al., 2011).

## Smart Scan of the volume: a solution for structures with complex cytoarchitecture

**Figure 1:**
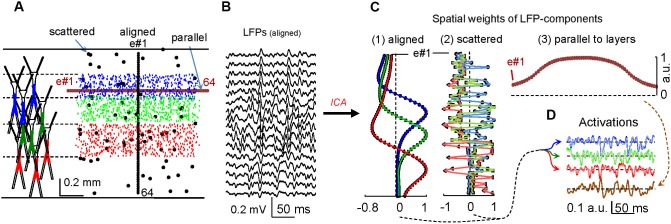
**Correct spatial sampling of electric fields optimizes the separation of synaptic contributions to LFPs in complex brain structures. (A)** Computational model of mulicompartmental units loosely arranged in a volume. The colored dendritic portions correspond to synaptically activated domains (left). Small dots indicate the position of the somata in the aggregate and the colors represent their activation by one of three different synaptic inputs. Larger dots indicate the position of 64 “recording” sites where the LFPs are estimated. Three different electrode configuration were used for the ICA: (1) aligned (cell axis), (2) parallel (cell body layers) and (3) scattered. **(B)** LFPs obtained at selected sites under configuration (1). **(C)** Spatial weight curves of the LFP components obtained by the ICA. Configurations that span vertically (1 and 2) yielded three LFP components with an identical temporal activation **(D)**, although only the aligned group offered a smooth curve matching the respective synaptic territories. Superimposed tracings in solid and feeble colors belong to ICA-derived and original activations, respectively. Recordings made parallel to the layers containing cell bodies (3) do not discriminate components as only one is obtained with a temporal activation that is a mix of all sources (in brown).

In the hippocampus, the use of linear silicon probes with recording sites aligned in parallel to the main cell axis provides laminar LFPs on account of the dominant flow of unitary currents in this direction. Hippocampal LFP generators have simple spatial distributions that follow the theoretical expectations of synaptic inputs to either planar or curved populations of neurons (Makarova et al., 2011; Fernández-Ruiz et al., 2013). In order to test ICA in more complex structures with no evident anatomical regularity, we modified our computational models to build a fake structure with loosely packed neurons (Figure 1). Three different populations were established functionally by implementing as many specific inputs to groups of neurons that had their somata located within contiguous bands, while maintaining a heavy overlap of their dendrites (see Figure 1A). The simultaneous activation of all three inputs with different time courses ensured a thorough blending of the inward and outward membrane currents within the volume.

In experimental situations there is a strong limitation on the placement of electrodes in suitable locations. This is important since electrodes should aim the sites where voltage gradients best describe the multiple activated synaptic territories. In the model, we tested three different spatial arrangements of “recording” sites (electrodes): one with sites scattered throughout the neural volume, and two linear arrays parallel and perpendicular to the layers containing the cell bodies (Figure 1A). Accordingly, we simulated three different spatial samplings of the same electrical field, and the LFPs “recorded” in each simulation were analyzed by the KD-ICA algorithm (Figure 1B; Chen, 2006). When the recording sites were situated over the direction parallel to the main cell axes (1 and 2 in Figure 1C), the ICA reflects the synaptic components with high temporal fidelity and each reproduced one of the original inputs (Figure 1D). However, when the LFPs are recorded in sites parallel to the cell body layers (3 in Figure 1C), the ICA fails to discriminate between inputs and it returns a single component with mixed temporal course. This happens because the impact of the currents generated by all synaptic inputs on each electrode has a similar weight.

It is noteworthy that in scattered and aligned cases, the number of components and their time courses were identical (Figures 1C, D). The main difference was in the corresponding spatial curves, which in the scattered case provides no clear anatomical information. Therefore, while knowing the site of the electrodes helps to identify the pathway responsible for the ICA-derived LFP components from the spatial curves, these are not relevant for their temporal definition and quantitative reliability, which remain intact. This is also relevant to complex structures, for which only a fraction of the recording sites may suffice as long as they cover enough portions of the respective spatial voltage shells produced by each of the co-activated pathways. We successfully used this procedure in experiments to minimize cross-contaminations (Benito et al., 2013). An additional advantage over common approaches to explore the cellular basis of LFPs, such as current source density analysis, is that the latter must be validated by a full spatial scan of the source and it renders a large proportion of spurious currents (see Fernández-Ruiz et al., 2012) due to the lack of a true baseline in AC-coupled recordings. By contrast, the ICA provides full temporal resolution as the temporal part of ICA components is insensitive to transient influences of co-activated sources that shall appear in other components (for a detailed comparison of these approaches, see Martín-Vázquez et al., 2013).

## A test in the lateral geniculate nucleus of the monkey

**Figure 2:**
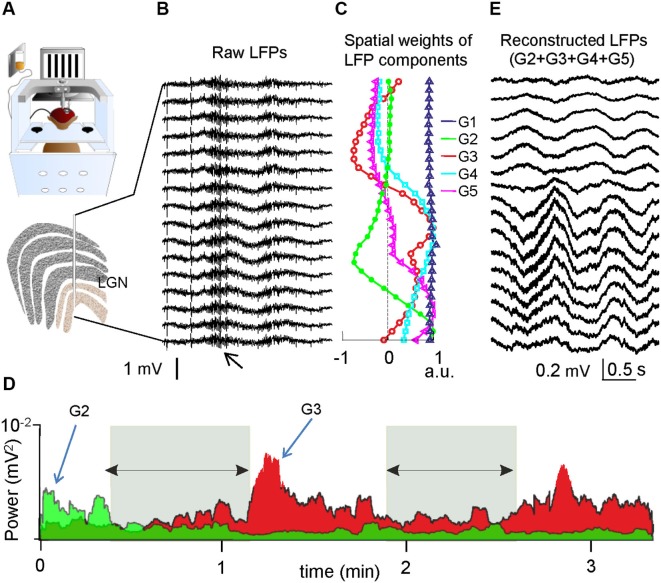
**LFP components in the monkey LGN. (A)** Recordings were obtained with a linear array throughout the LGN while the animal performed a visual task. **(B)** Sample epoch of simultaneous LFPs. The high frequency bouts (small arrow) belong to 50 Hz noise during facial muscle movements. **(C)** Spatial weights of the ICA-derived components. G1 (blue) entered with similar power to all electrodes, thus it belongs to a remote source. G2-G5 have maxima in different layers within the recording area, indicating different afferent pathways with local synaptic territories. **(D)** Evolution of the power in two LFP components (G2 and G3) in a sample epoch. The activity is specifically altered in some but not all components according to behavioral states. The periods marked by the horizontal arrows coincide with eye closure and somnolence. **(E)** Virtual LFPs can be reconstructed for a desired component or group, enabling close examination and quantitative use of the temporal details. These results were obtained by analyzing data in earlier studies (Rivadulla et al., 2012).

It might therefore be anticipated that the ICA-approach is capable of revealing reliable LFP components no matter how complex the spatial organization of the units and population, as long as it efficiently separates the spatial and temporal features of the sources. To demonstrate this inference, we examined LFPs recorded with a linear array in the lateral geniculate nucleus (LGN) of a monkey (*Macaca mulatta*) trained to perform a visual task (Figure 2; Rivadulla et al., 2012). Though this nucleus has six well-defined layers of cell bodies (Figure 2A), most neurons have a multipolar near-radial symmetry that is unsuitable for LFP contribution, while others show a suitable axial symmetry but their dendrites may remain in one layer or extend across several (e.g., Wilson and Hendrickson, 1981), as in the former model. Preliminary data showed that raw LFPs are very similar along the recording track indicating a dominant remote contribution (Figure 2B). The ICA revealed five stable components (Figure 2C). As expected, one component (G1, blue in Figure 2C) has a linear spatial profile corresponding to the contribution of a remote source lying outside the zone spanned by the electrodes (the distant sources have a similar impact on all the electrodes). The other four components (G2–G5) have smooth spatial curves and maxima at different loci. As suggested by their distinct time courses and specific state-dependence, these components most likely describe synaptic activity elicited by afferent input from different origins, such as the retina, the cortex, the nucleus reticularis or the midbrain (Sherman and Guillery, 2001). For instance, short periods of somnolence when the animal closed its eyes produced variations in the power of some LFP components but not others (Figure 2D). Furthermore, while the temporal details that can be appreciated by the naked eye in raw LFPs, mostly reflecting the activity in the strong remote generator, the ICA allows virtual LFPs of one or a group of components to be reconstructed, thus, the fluctuations of local generators become visible (Figure 2E).

## Conclusion

The difficulties in understanding LFPs are not derived from a lack of theoretical knowledge but rather, they are the product of technical limitations. Whenever a sizable LFP is recorded there must be a spatial segregation of the inward and outward currents within the domains of individual neurons, and a spatial organization of the activated population that makes their extracellular summation possible. Otherwise, the positive and negative currents cancel each other out and LFPs are not apparent. One may imagine different architectures in which currents elicited by inputs from different pathways may be cancelled out in some regions but not in others (Fernández-Ruiz et al., 2013). Hence, it is important to understand the geometry and arrangement of cells, and of their inputs. However, even if the spatial distribution of ICA-components turns out not to be informative, the approach is not invalidated. Indeed, the temporal part of the ICA-derived LFP components may still be a reliable readout of activity in specific afferent populations and it can be used to quantitatively evaluate their global output in different epochs and behavioral states.

## Conflict of interest statement

The authors declare that the research was conducted in the absence of any commercial or financial relationships that could be construed as a potential conflict of interest.
